# The Effect of Chronic Immunosuppressive Regimen Treatment on Apoptosis in the Heart of Rats

**DOI:** 10.3390/ph17091188

**Published:** 2024-09-10

**Authors:** Anna Surówka, Michał Żołnierczuk, Piotr Prowans, Marta Grabowska, Patrycja Kupnicka, Marta Markowska, Zbigniew Szlosser, Karolina Kędzierska-Kapuza

**Affiliations:** 1 Department of Plastic, Endocrine and General Surgery, Pomeranian Medical University, 72-010 Szczecin, Polandzbigniew.szlosser@pum.edu.pl (Z.S.); 2 Department of Vascular Surgery, General Surgery and Angiology, Pomeranian Medical University, 70-111 Szczecin, Poland; 3 Department of Histology and Developmental Biology, Faculty of Health Sciences, Pomeranian Medical University, 70-111 Szczecin, Poland; marta.grabowska@pum.edu.pl; 4 Department of Biochemistry and Medical Chemistry, Pomeranian Medical University, 70-111 Szczecin, Poland; patrycja.kupnicka@pum.edu.pl; 5 Department of Plastic and Reconstructive Surgery, 109 Military Hospital, 71-422 Szczecin, Poland; markowskamh@gmail.com; 6 Department of Gastroenterological Surgery and Transplantology, National Medical Institute, Ministry of Interior Affairs and Administration, 02-507 Warsaw, Poland; karolina.kedzierska@gmail.com

**Keywords:** immunosuppressive drugs, calcineurin inhibitors, tacrolimus, mycophenolate mofetil, heart failure, apoptosis, caspases

## Abstract

Chronic immunosuppressive therapy is currently the only effective method to prevent acute rejection of a transplanted organ. Unfortunately, the expected effect of treatment brings a number of grave side effects, one of the most serious being cardiovascular complications. In our study, we wanted to investigate how treatment with commonly used immunosuppressive drugs affects the occurrence of programmed cardiac cell death. For this purpose, five groups of rats were treated with different triple immunosuppressive regimens. Cardiac tissue fragments were subjected to the TUNEL assay to visualize apoptotic cells. The expression of Bcl-2 protein, Bax protein, caspase 3 and caspase 9 was also assessed. This study indicates that all immunosuppressive protocols used chronically at therapeutic doses result in an increased percentage of cells undergoing apoptosis in rat heart tissue. The greatest changes were recorded in the TMG (rats treated with tacrolimus, mycophenolate mofetil and glucocorticosteroids) and CMG (rats treated with cyclosporin A, mycophenolate mofetil and glucocorticosteroids) groups. The TRG (rats treated with rapamycin, tacrolimus and glucocorticosteroids) group showed the lowest percentage of apoptotic cells. The internal apoptosis pathway was confirmed only in the TMG group; in the remaining groups, the results indicate programmed cell death via the receptor pathway.

## 1. Introduction

Organ transplantation is an increasingly safe and common treatment for the end-stage and irreversible failure of vital internal organs, such as the kidney, liver, heart or lung [1,2]. Along with the number of procedures performed, the number of patients undergoing long-term immunosuppressive treatment, the aim of which is to inhibit or reduce the recipient’s immune response to antigens of the transplanted organ, is increasing [3,4]. Modern immunosuppressive treatment regimens reduce the risk of acute graft rejection and provide one-year graft survival at a very high level [5]. This is currently the only effective therapy used in transplantology to enable the long-term and normal function of the received organ. The use of immunosuppressive drugs is also associated with a number of side effects, such as infections, malignancies, neurotoxicity, hepatotoxicity or nephrotoxicity [3,6]. Nevertheless, cardiovascular diseases (CVDs) are the main cause of death in patients after kidney transplantation, thus becoming the most serious complication for organ recipients [7,8]. The underlying disease, which is renal failure, increases the risk of cardiovascular events by its very nature; however, compared to the entire population, kidney recipients are still the most vulnerable group of patients [9]. Immunosuppressive therapy may trigger metabolic syndrome, causing hyperlipidemia, disorders of carbohydrate metabolism, diabetes, hypertension or fibrinolysis disorders [7,10,11,12]. In addition, it leads to myocardial hypertrophy and fibrosis, arrhythmias and atherosclerosis of blood vessels. What is more, undesirable effects of treatment may contribute to the development of cardiovascular diseases [8,13,14].

Apoptosis is a highly controlled process of programmed death of damaged, abnormal or worn-out cells. Under physiological conditions, this mechanism is responsible for proper development and tissue remodeling [15,16]. The apoptosis process can also be initiated by factors such as oxidative stress, mechanical damage to the cell, ionizing radiation or cytotoxic drugs or in response to cytokines [17,18]. Factors that impair the functioning of the heart trigger compensatory mechanisms, which, in the initial phase, ensure proper blood supply to the body. However, chronic strain on the heart leads to the development of heart failure [19]. Current research indicates that apoptosis of cardiac myocytes is one of the most important mechanisms inducing the development of cardiac dysfunction. In addition, programmed cell death contribute to acute and chronic myocardial ischemia, dilated cardiomyopathies or arrhythmias. It has also been confirmed that, in a transplanted heart, apoptosis occurs with a higher frequency than in a healthy heart [20,21,22,23,24].

Optimizing immunosuppression means finding a balance between efficacy and patient safety. Therefore, efforts should be made to identify the direct mechanisms affecting the normal structure and function of the cardiovascular system, as well as the role of individual treatment regimens in these processes. The main objective of this study was to investigate whether long-term treatment of rats with commonly used three-drug immunosuppressive regimens affected the occurrence of apoptosis in the myocardium, and, as such, whether it could affect the development of heart failure.

The presented results are a continuation of our previous studies showing the effect of immunosuppressive treatment on morphological changes in the heart and abdominal aorta of rats. Additionally, we noted changes in the expression of metalloproteinase-2, metalloproteinase-9 and their specific inhibitors. The research work was preceded by a thorough review of the literature. [13,25,26,27].

## 2. Results

### 2.1. Bax Protein Expression

**Figure 1:**
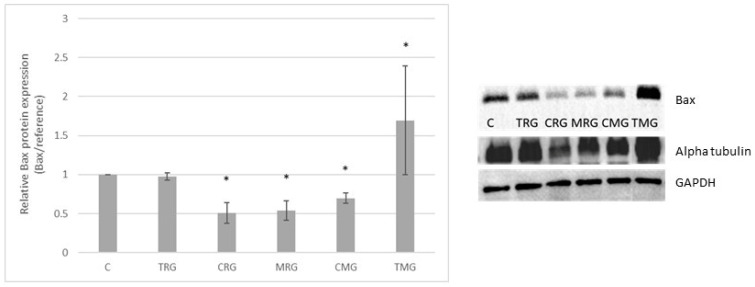
Representative Western blots and densitometric analysis of Bax protein expression levels (normalized to reference protein) in hearts of C—control group without any medication; TRG—rats treated with rapamycin, tacrolimus, glucocorticosteroids; CRG—rats treated with rapamycin, cyclosporin A, glucocorticosteroids; MRG—rats treated with rapamycin, mycophenolate mofetil, glucocorticosteroids; CMG—rats treated with cyclosporin A, mycophenolate mofetil and glucocorticosteroids; TMG—rats treated with tacrolimus, mycophenolate mofetil and glucocorticosteroids; the results are expressed as means ± SD. * *p* < 0.01 (Mann–Whitney U test).

Western-blot-determined Bax protein expression was 60% lower than in the control in CRG, 55% lower in MRG and 35% in CMG groups (*p* < 0.01). In the TMG group, the expression of Bax protein significantly increased by 68% in comparison with the control group (C) (*p* < 0.01). The expression in the TRG group did not change. Expression of Bax protein is presented in Figure 1.

### 2.2. Bcl-2 Protein Expression

**Figure 2:**
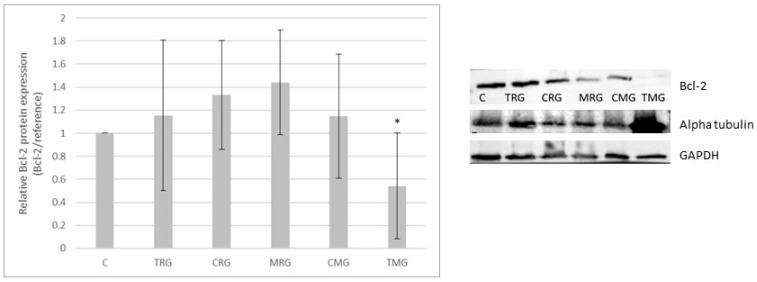
Representative Western blots and densitometric analysis of Bcl-2 protein expression levels (normalized to reference protein) in hearts of C—control group without any medication; TRG—rats treated with rapamycin, tacrolimus, glucocorticosteroids; CRG—rats treated with rapamycin, cyclosporin A, glucocorticosteroids; MRG—rats treated with rapamycin, mycophenolate mofetil, glucocorticosteroids; CMG—rats treated with cyclosporin A, mycophenolate mofetil and glucocorticosteroids; TMG—rats treated with tacrolimus, mycophenolate mofetil and glucocorticosteroids; the results are expressed as means ± SD. * *p* < 0.05 (Mann–Whitney U test).

Western-blot-determined Bcl-2 protein expression was 15% higher than in the control group (C) in TRG, 33% in CRG, 44% in MRG and 15% in CMG groups; however, the difference was not statistically significant. The TMG group was characterized by statistically decreased Bcl-2 protein expression when compared to the control group (*p* < 0.05) by 45%. Expression of Bcl-2 protein is presented in Figure 2.

### 2.3. Caspaze 3 Expression

**Figure 3:**
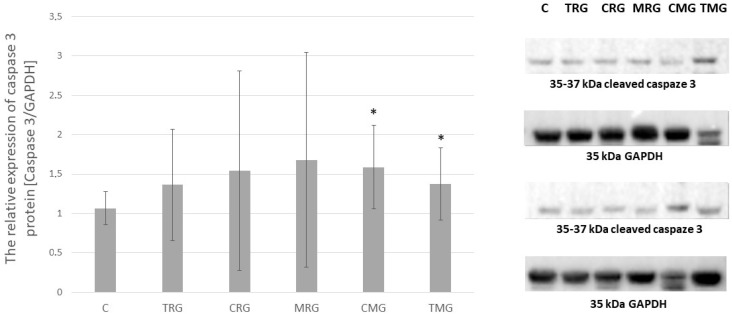
Representative Western blots and densitometric analysis of caspase 3 protein expression levels (normalized to GAPDH) in hearts of C—control group without any medication; TRG—rats treated with rapamycin, tacrolimus, glucocorticosteroids; CRG—rats treated with rapamycin, cyclosporin A, glucocorticosteroids; MRG—rats treated with rapamycin, mycophenolate mofetil, glucocorticosteroids; CMG—rats treated with cyclosporin A, mycophenolate mofetil and glucocorticosteroids; TMG—rats treated with tacrolimus, mycophenolate mofetil and glucocorticosteroids; the results are expressed as means ± SD. * *p* < 0.05 (Mann–Whitney U test).

The expression of caspase 3 was, on average, elevated in every group compared to the control group. The statistical significance was observed only in CMG and TMG groups, *p* < 0.05, where the expression of caspase 3 was 49% and 29% higher, respectively, than in the control group. The results are shown in Figure 3.

### 2.4. Caspaze 9 Expression

**Figure 4:**
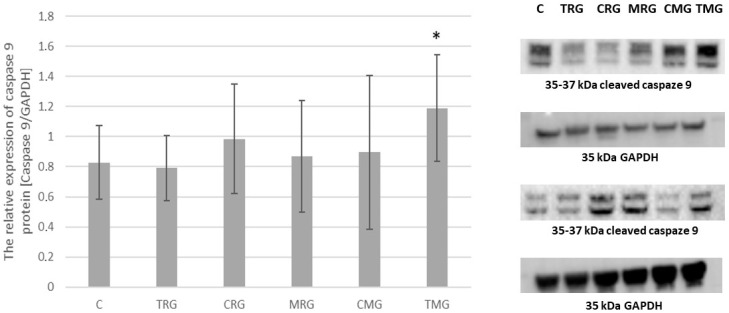
Representative Western blots and densitometric analysis of caspase 9 protein expression levels (normalized to GAPDH) in hearts of C—control group without any medication; TRG—rats treated with rapamycin, tacrolimus, glucocorticosteroids; CRG—rats treated with rapamycin, cyclosporin A, glucocorticosteroids; MRG—rats treated with rapamycin, mycophenolate mofetil, glucocorticosteroids; CMG—rats treated with cyclosporin A, mycophenolate mofetil and glucocorticosteroids; TMG—rats treated with tacrolimus, mycophenolate mofetil and glucocorticosteroids; the results are expressed as means ± SD. * *p* < 0.05 (Mann–Whitney U test).

The expression of caspase 9 was elevated in CRG, MRG, CMG and TMG groups compared to the control group. However, statistical significance was observed only in the TMG group, *p* < 0.05, where the level of caspase 9 was 44% higher than the control group. The results are shown in Figure 4.

### 2.5. TUNEL Assay

**Figure 5:**
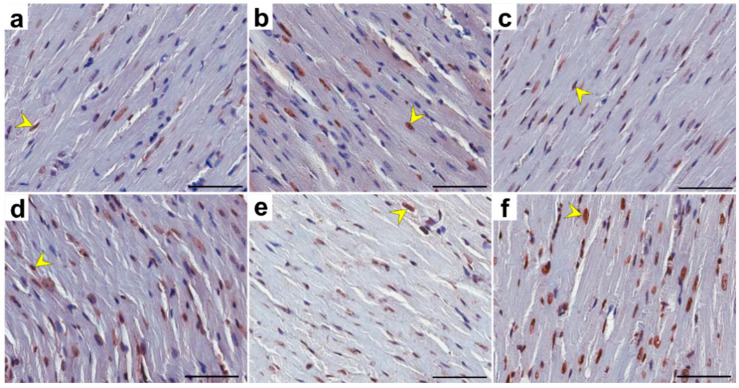
Representative light micrographs of the TUNEL-positive cells in the heart of rats in the control (**a**), TRG (**b**), CRG (**c**), MRG (**d**), CMG (**e**), TMG (**f**) groups. TUNEL-positive cells with brown-stained nuclei (yellow arrowheads) were observed. C—control group without any medication; TRG—rats treated with rapamycin, tacrolimus, glucocorticosteroids; CRG—rats treated with rapamycin, cyclosporin A, glucocorticosteroids; MRG—rats treated with rapamycin, mycophenolate mofetil, glucocorticosteroids; CMG—rats treated with cyclosporin A, mycophenolate mofetil and glucocorticosteroids; TMG—rats treated with tacrolimus, mycophenolate mofetil and glucocorticosteroids;. TUNEL—terminal deoxynucleotidyl transferase-mediated dUTP nick end-labeling. Scale bar: 50 µm.

In the control and in the experimental groups of rats, TUNEL-positive cells (with nuclear DNA fragmentation) in the heart tissue were characterized by brown-stained nuclei of cardiomyocytes (Figure 5). In TRG, CRG, MRG, CMG and TMG groups, the percentage of TUNEL-positive cells was significantly higher (*p* < 0.001 and *p* = 0.003, respectively) than that in the control group (Table 1).

## 3. Discussion

**Figure 6:**
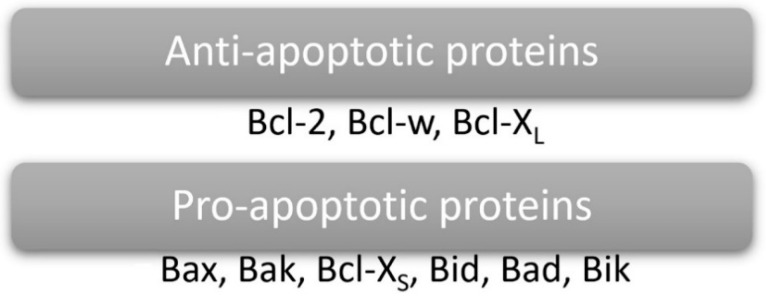
Bcl-2 family proteins that inhibit and activate apoptosis.

Apoptosis is an active physiological process leading to suicidal cell death in response to abnormal signals from the external environment or defects in the structure and function of the cell itself [15]. The process is regulated by a number of diverse proteins, among which caspase family enzymes play a significant role. Their main task is the degradation of structural and enzymatic proteins, which ultimately leads to the destruction of the entire cell. Due to their role, we distinguish activator caspases (-2, -8, -9, -10, -12) and executive caspases (-3, -6, -7) [28,29,30]. Apoptosis can be initiated in several ways, with two main ones coming to the foreground here, namely the intrinsic and extrinsic pathway. The internal (mitochondrial) pathway is triggered by, e.g., factors such as oxidative stress, physical cell damage, pathogens, ionizing radiation or cytotoxic drugs. Numerous proteins from the Bcl-2 family are involved in the regulation of this pathway. Due to their activity and structure, they have been divided into two groups: anti-apoptotic-blocking cell death and pro-apoptotic-stimulating cell death (Figure 6) [17,18,31,32,33].

Mitochondria are the starting point for the internal apoptosis pathway, where a complex of p53 proteins with Bcl-2/Bcl-XL proteins is formed under the influence of stress factors. Anti-apoptotic proteins become bound and pro-apoptotic proteins such as Bid and Bax oligomerize to form pores in the mitochondrial membrane. As a result, cytochrome c is released into the cytoplasm, where it binds to the Apaf-1 factor and caspase 9, thus forming the apoptosome. The Bcl-2/Bcl-XL proteins are inactivated by binding to the Bid, Bad, Bik and Bak proteins on the surface of the apoptosome. The whole process leads to the activation of caspase 3. In the context of the intrinsic pathway of apoptosis, the most commonly studied proteins are Bcl-2 and Bcl-X_L_; however, the imbalance of the expression of anti-apoptotic proteins in favor of pro-apoptotic proteins determines the final death of the cell [34,35,36,37]. The extrinsic (receptor) pathway is associated with DR death receptors, which consist of receptors for TNF-α, FasL and TRAIL. The receptor also has an intracellular DD (death domain) that binds Fas-associated death domain (FADD) adaptor proteins in response to ligand attachment to one of the DR receptors. The attachment of procaspase 8 or 10 results in the formation of a death-inducing signaling complex (DISC). In type I cells, DISC activates caspase 8, thereby initiating activation of effector caspase 3, followed by apoptosis. In type II cells, the mitochondrial pathway is indirectly mobilized. Caspase 8 causes proteolysis of Bid protein to the active form t-Bid and its movement into the mitochondrion. This stimulates Bcl-2 family proteins, which integrate into the outer membrane and release cytochrome c [15,18,38,39].

**Figure 7:**
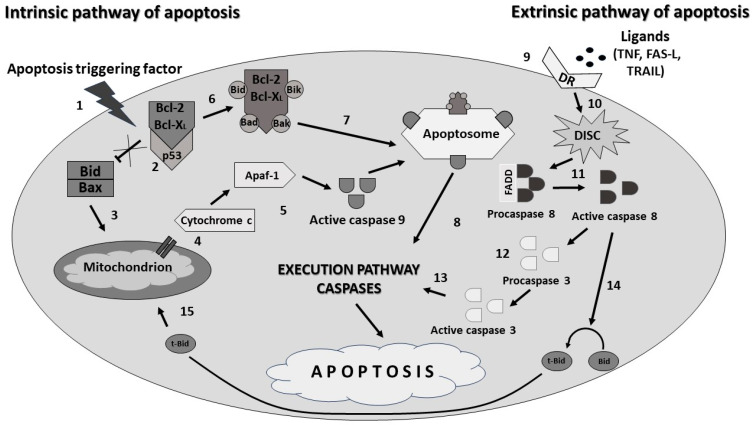
Simplified scheme of the mechanism of apoptosis by the extrinsic and intrinsic pathways. **1.** Initiation of the intrinsic pathway of programmed cell death. **2.** Fusion of p53 protein with Bcl-2 and Bcl-X_L_ proteins. **3.** Pro-apoptotic proteins creating pores in the membrane of mitochondrion. **4.** Cytochrome c releasing. **5.** Cytochrome c binds with apoptotic protease activating factor 1 (Apaf-1) and caspase 9 to create an apoptosome. **6.** Fusion of a complex of bound anti-apoptotic proteins with Bid, Bik, Bad, Bak proteins. **7.** Attachment of the complex to the apoptosome. **8.** The active complex induces the execution caspase cascade, leading to programmed cell death. **9.** Pro-apoptotic ligands bind to the death receptor (DR). **10.** Creation of the death-inducing signaling complex (DISC). **11.** Formed DISC binds Fas-associated death domain (FADD), leading to caspase 8 activation. **12**. Activation of caspase 3. **13.** Induction of the execution caspase pathway and apoptosis. **14.** Proteolysis of Bid protein to the active form t-Bid. **15.** Active t-Bid protein migration to mitochondrion.

A simplified diagram of the mechanism of apoptosis is presented in Figure 7.

The mechanism of action of drugs from the calcineurin inhibitor (CNI) group involves the inhibition of cellular calcineurin, or serine-threonine phosphatase, whose main function is to activate T cells. When an antigen is recognized by T cell receptors, calcium ions are released from the endoplasmic reticulum and a calmodulin–calcineurin–Ca^2+^ complex is formed. Activation of serine/threonine phosphatase results in the dephosphorylation of nuclear-factor-activated T cells (NFATcs), which induce the transcription of genes for many cytokines. The released interleukins activate the processes of leukocyte proliferation and differentiation. Calcineurin inhibitors show high affinity for immunophilins, specific cytoplasmic receptors. Cyclosporine (CsA) readily penetrates the cell and binds to cyclophilin A. Tacrolimus (TAC) binds to the immunophilin FK-BP12 in the cell cytoplasm. In both cases, the resulting complexes selectively inhibit calcineurin, thereby reducing the formation of IL-2, IL-3, IL-4, TNF-α, IFN-γ, G-CSF, M-CSF [40,41]. The mTOR protein is an intracellular serine/threonine kinase that affects the processes of protein transcription and translation and regulates cell division. Rapamycin, like tacrolimus, binds to the cytoplasmic protein FKBP-12. The resulting complex inhibits mTOR kinase. The cell’s specific signal transduction pathways are blocked, preventing the translation of proteins involved in the cell cycle. Lymphocytes remain in the G1 phase and cytokine-dependent cell proliferation and immunoglobulin production are inhibited. The immunosuppressive effect of mTOR inhibitors is weaker than that of calcineurin inhibitors [42,43]. Mycophenolate mofetil (MMF) is a selective and reversible inhibitor of inosine monophosphate dehydrogenase, which is required for the de novo synthesis of purines and guanosine nucleotides. For lymphocytes, the de novo pathway is the only known pathway for purine synthesis; hence, MMF exhibits potent activity against this group of cells. The cytotoxicity of the cell division inhibitor significantly reduces the proliferation of T and B lymphocytes and, to a lesser extent, inhibits the subdivisions of fibroblasts, monocytes and smooth myocytes. Unlike CNI and mTOR inhibitors, mycophenolate mofetil does not inhibit cytokine synthesis [44,45,46].

In the course of this study, it was observed that there occurred a statistically significant increase in apoptosis in the cardiac tissues of rats receiving each of the immunosuppressive treatment regimens administered. The percentage of TUNEL-positive cells, compared to the control group, was the highest in the TMG group, while it was the lowest in the TRG group. Nevertheless, all immunosuppressive drug sequences used in the study caused increased cell death by apoptotic death. The triple-drug regimens of CRG, MRG and CMG, compared to the control group, decreased the expression of the pro-apoptotic protein Bax associated with the intrinsic pathway. There were no significant changes in the expression of this protein in the TRG group. The results presented here may indicate that the above drug sequences do not activate programmed cell death in the rat heart via the mitochondrial pathway, as the expression of Bax protein remained equal to or lower than in the control group. The TMG treatment regimen was the only one to significantly increase the level of this protein. There was a 68% increase in Bax expression in this group, which may suggest the occurrence of apoptosis via the intrinsic pathway with this treatment. Expression of the anti-apoptotic protein Bcl-2 in rat cardiac tissue increased with four drug regimens—TRG, CRG, MRG and CMG. The changes were not statistically significant compared to the control group. Summarizing the results obtained in the above-mentioned groups, the predominance of inhibitory factors over factors promoting apoptosis through the intrinsic pathway can be stated. On the other hand, a statistically significant decrease in Bcl-2 expression was noted in the cardiac tissue of rats treated with the TMG regimen, which, together with the above-described increase in pro-apoptotic protein expression, may confirm the occurrence of apoptosis by the mitochondrial pathway in this study group. The expression of effector caspase 3 was increased in all study groups; however, only in the CMG and TMG groups was the increase statistically significant. The obtained results correlate with the visualized highest number of TUNEL-positive cells in the rat heart after both treatment regimens. A statistically significant increase in caspase 9 was noted only in the TMG group, which, in combination with the described results of Bcl-2 family protein expression, also supports an intrinsic apoptosis pathway after the TMG treatment regimen. In the available literature, we can only find reports on the effect of single immunosuppressive drugs on the occurrence of apoptosis in cardiac tissue, which makes it very difficult for us to compare our results with other studies. There is also a lack of clear information as to the pathway of programmed cell death used in response to the drugs that are the subject of our study.

Chen et al. pointed out the interdependence of the occurrence of apoptosis in rat cardiac cells and on the dose of cyclosporin A administered. They treated isolated cardiomyocytes with either hydrogen peroxide or hypoxia, thereby exposing them to induced oxidative stress. Cyclosporin A was also added to individual cell lines at concentrations ranging from 0.01 to 10 µM. The number of cells undergoing apoptosis, as in our work, was measured using the TUNEL method. Oxidative stress alone caused an increased percentage of cells undergoing apoptotic death. Cyclosporin A at low concentrations reduced the occurrence of programmed cell death induced by oxidative stress while, at higher concentrations (10 µM), it intensified this phenomenon [47]. Calcineurin inhibitors reduce cytokine expression, inhibit T-cell proliferation and activity and thus can reduce inflammation in response to a strong stress stimulus [48,49,50]. The use of higher doses or chronic use of these drugs may be associated with their toxic effects on cells. All of the three-drug protocols used in our study, which included cyclosporine, increased the percentage of cardiomyocytes undergoing apoptosis. The administered dose, 5 mg/kg/d, corresponds to the therapeutic dose; thus, it may be a toxic amount for myocardial cells and may lead to programmed cell death, especially with prolonged use.

The study by Chua et al. also observed the effect of a calcineurin inhibitor on myocardial cells subjected to ischemia. The study was conducted on a small pig model that had its left anterior descending artery surgically ligated, thereby inducing myocardial infarction. Some of the animals received an intra-arterial dose of tacrolimus mixed in the range of 0.25 mg^−1^ mg before surgery. It was noted that the addition of tacrolimus reduced infarct-induced oxidative stress. There was an increase in the expression of the anti-apoptotic protein Bcl-2 and a significant decrease in the expression of the pro-apoptotic protein Bax in the group of pigs that received tacrolimus prior to infarction induction compared to the group in which the infarction was induced without administering the drug. A reduced amount of cell nuclei disintegration was also noted in this group. The results indicate a protective effect of tacrolimus in the course of acute myocardial ischemia [51]. As in the previous study, the researchers used a low, single dose of the calcineurin inhibitor. During our experiment, rats received chronic treatment and a many-times-higher dose of tacrolimus (4 mg/kg/d). All of the three-drug protocols based on the use of tacrolimus promoted increased apoptosis, while, in the TMG protocol, cell death occurred via an intrinsic pathway activation mechanism.

The use of mycophenolate mofetil on a rat model subjected to induced myocardial ischemia also reduced the area affected by infarction. The drug’s administration contributed to an increase in Bcl-2 protein expression and a decrease in Bax protein expression in cardiac tissue compared to the group of rats after an episode of ischemia alone. Mycophenolate mofetil also contributed to a decrease in the expression of TNFα, which is a ligand for death receptors in the extrinsic pathway of apoptosis [52]. The dose of the drug used in this study (20 mg/kg/d) corresponded to the dose used in our experiment; however, as in other studies, it was a single dose. The process of ischemia itself caused an increase in the expression of TLR4, NF-κB and Bax and a decrease in Bcl-2 levels, thereby contributing to increased apoptosis in the rat heart. Mycophenolate mofetil reduced the body’s inflammatory response to infarction, thereby reducing programmed cardiomyocyte death. Our study was conducted on healthy rats receiving immunosuppressive treatment for 6 months. Mycophenolate mofetil is a drug that reduces the proliferation of T and B lymphocytes, fibroblasts and monocytes and reduces oxidative stress in the course of ongoing inflammation. Thus, it may reduce the frequency of apoptosis in response to a strong stress stimulus such as ischemia [44,45]. The results of our study indicate that the chronic intake of this antimetabolite or use in combination with other immunosuppressive drugs may abrogate its protective effects or even adversely affect cells.

The results presented are preliminary to further studies on the effects of chronic immunosuppressive treatment on the development of heart failure in organ recipients. Nevertheless, demonstrating the phenomenon of apoptosis with each of the available treatment protocols is an important observation. In this study, we have also demonstrated the pathways of apoptosis activation that were triggered during the drug treatment of rats. Current forms of chronic cancer therapy involve the use of BH3-mimetic drugs, which are designed to activate apoptosis by inhibiting anti-apoptotic BCL-2 group proteins [53]. It is hoped that, in the future, we will achieve knowledge of apoptosis suppression in patients with increased cardiovascular risk. Another important aspect is the study of factors affecting the phenomenon of programmed cell death with immunosuppressive drugs, such as the level of oxidative stress and expression of TNF-alpha, interleukins and chemokines [23,54,55]. In addition to pharmacological methods, attention is increasingly being drawn to at least the use of natural medicine in leveling apoptosis-inducing factors in the cardiovascular system [56]. A strategy based on supporting the body can improve prognosis and reduce the negative effects of therapy. The correct selection of an immunosuppressive regimen means finding a balance between efficacy and patient safety. Further research is needed to identify the direct mechanisms that affect the proper structure and function of the cardiovascular system, as well as the role of individual treatment regimens in these processes.

## 4. Materials and Methods

### 4.1. Animals

This study was conducted on the hearts of rats obtained from an experiment carried out by Kedzierska et al. [57]. Part of the collected tissue fragments was secured in a liquid nitrogen vat and then frozen at −86 °C. Part of the research material was fixed in 4% paraformaldehyde and then immersed in paraffin blocks.

**Figure 8:**
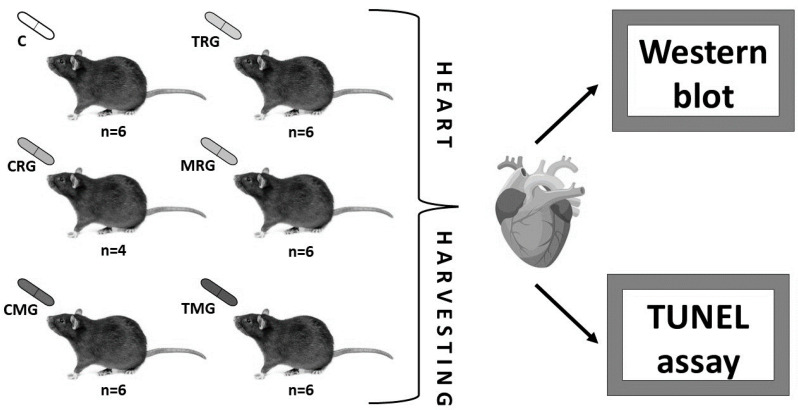
Experimental protocol of drugs used in the current research: C—control group without any medication; TRG—rats treated with rapamycin, tacrolimus, glucocorticosteroids; CRG—rats treated with rapamycin, cyclosporin A, glucocorticosteroids; MRG—rats treated with rapamycin, mycophenolate mofetil, glucocorticosteroids; CMG—rats treated with cyclosporin A, mycophenolate mofetil, and glucocorticosteroids; TMG—rats treated with tacrolimus, mycophenolate mofetil, and glucocorticosteroids; n—number.

The experiment was approved by the Local Ethical Committee for Experiments on Animals (Decision No. 24/2008 of 24 November 2008). The study was conducted on 36 male Wistar rats with an average body weight of 305 g. The animals were divided into six groups: a control group (C) and five experimental groups. Rats from particular experimental groups received different regimens of immunosuppressive drugs, according to the most commonly used models of treatment of organ recipients (Figure 8). Rats in the control group did not receive any drugs.

The medications in the experiment were administered orally in the following doses: tacrolimus (Prograf; Astellas Pharma Inc., Tokyo, Japan)—4 mg/kg/day, cyclosporine A (Sandimmun-Neoral; Novartis International AG, Basel, Switzerland)—5 mg/kg/day, mycophenolate mofetil (Cellcept; Hoffman-La Roche Ltd., Basel, Switzerland)—20 mg/kg/day, rapamycin (Rapamune; Pfizer, Inc., New York, NY, USA)—0.5 mg/kg/day, prednisone (Encorton; Polfa, Pabianice, Poland)—4 mg/kg/day. Animals were given medication once a day for 6 months. Three months into the experiment, the dose of drugs was increased adequately with respect to the increase in body weight of the rats. Two rats from the CRG group (rats treated with rapamycin, cyclosporin A, glucocorticosteroids) died during the 4th month of the study. After half a year of the experiment, the animals were euthanized by administration of ketamine hydrochloride at a dose of 50 mg/kg and necropsied with subsequent organ harvesting.

The standard post-transplant immunosuppressive treatment regimen involves the use of a calcineurin inhibitor, tacrolimus or cyclosporin A, combined with mycophenolate mofetil and a glucocorticosteroid. It is acceptable to replace one of the above-mentioned drugs with an mTOR inhibitor, rapamycin [58]. In our study, we used all possible drug combinations, using tacrolimus, cyclosporine, mycophenolate mofetil and rapamycin, which is the main difference in treatment groups. A constant component of all treatment regimens was the glucocorticosteroid, prednisone. Drug doses were based on available literature data [59,60,61,62,63]. As an approximation, we can assume that 6 months of a rat’s life is equivalent to 12 years of a human’s life [64].

### 4.2. Methods

#### 4.2.1. Western Blot Analysis

Tissue homogenates were treated with RIPA (radioimmunoprecipitation assay) lysis buffer containing protease and phosphatase inhibitors (cOmplete™, Mini Protease Inhibitor Cocktail, Roche, Switzerland, PhosSTOP™, Roche, Switzerland). Protein concentration in the filtrate was determined with the BCA (bicinchoninic acid) method using a commercial Pierce™ BCA Protein Assay Kit (Thermo Fisher Scientific™, Waltham, MA, USA). Then, electrophoretic protein fractionation was conducted using 14% polyacrylamide gel by placing 30 μg protein/well. The fractionated proteins were transferred onto a 0.2 μm PVDF (polyvinylidene difluoride) membrane (Thermo Fisher Scientific™, Waltham, MA, USA) by a wet transfer. Prior to incubation with antibodies, the membranes were placed in a blocking buffer—5% BSA (bovine serum albumin) (Merck, Burlington, MA, USA) for 60 min. The protein expression was detected with the use of an antibody against BCL-2 (ab196495) (Abcam, Cambridge, UK) and Bax (ab216494) (Abcam, Cambridge, UK), caspase 3 (ab184787) (Abcam, Cambridge, UK) and caspase 9 (ab184786) (Abcam, Cambridge, UK) diluted 1:500 and sAb goat anti-rabbit/mouse IgG HRP H&L (ab6789, ab205718) (Abcam, Cambridge, UK). Expression of reference proteins, GAPDH (ab181602) (Abcam, Cambridge, UK) and/or alpha-tubulin (ab7291) (Abcam, Cambridge, UK), was detected. The membranes were developed with an ECL (enhanced chemiluminescence) Advance Western Blotting Detection Kit (GE Healthcare, Chicago, IL, USA), and, subsequently, bands were visualized using the Molecular Imager ChemiDock XRS+ (Bio-Rad, Hercules, CA, USA). Quantitative analysis was performed using Image Lab Software 6.1 (Bio-Rad, Hercules, CA, USA). The density of the loading control (GAPDH and/or alpha-tubulin) was used to normalize the density of the analyzed proteins (BCL-2, Bax, caspase 3, caspase 9). The band with the highest intensity was used as a reference band. The normalization factor was calculated by dividing the signals from the housekeeping protein by the highest obtained value. Next, each band was divided by the corresponding normalization factor.

##### Statistical Analysis for the Western Blot Analysis

The results were statistically analyzed with Statistica 13 software and are presented as mean values ± SD. The Shapiro–Wilk W test did not show conformity with the normal distribution; the non-parametric Mann–Whitney U test was used for comparison of the groups. Statistical significance was set at *p* < 0.05.

#### 4.2.2. TUNEL Assay Result

A terminal deoxynucleotidyl transferase dUTP nick end-labeling (TUNEL) assay was carried out according to the manufacturer’s guidelines (ApopTag^®^ Peroxidase In Situ Apoptosis Detection Kit; Millipore, Billerica, MA, USA) to detect nuclear DNA fragmentation related to apoptosis. The heart sections were deparaffinized, rehydrated and digested with proteinase K (Dako, Glostrup, Denmark). The activity of endogenous peroxidase was blocked by the application of peroxidase blocking solution (Dako, Glostrup, Denmark). Next, the slides were incubated with terminal deoxynucleotidyl transferase (TdT; Millipore, Billerica, MA, USA) for 60 min in a humid chamber at 37 °C. Subsequently, the slides were incubated with the anti-digoxigenin antibody conjugated with peroxidase for 30 min in a humid chamber. In order to visualize the reaction, diaminobenzidine (DAB; Dako, Glostrup, Denmark) was used. The sections were counterstained with Mayer’s hematoxylin and then dehydrated and coverslipped. The sections were examined in a light microscope (Olympus BX 41, Hamburg, Germany).

##### Quantitative Analysis of TUNEL-Positive Cells

Slides with TUNEL-immunostained heart cross-sections were scanned at a magnification of 200× (resolution of 0.25 μm/pixel) with the ScanScope AT2 scanner (Leica Microsystems, Wetzlar, Germany). The obtained digital images were analyzed on the computer screen using the ImageScope viewer (Version 11.2.0.780; Aperio Technologies, Vista, CA, USA).

For the quantitative analysis of TUNEL-positive cells, a nuclear v9 algorithm (version 9.1; Aperio Technologies, Vista, CA, USA) was used. The areas for analysis were manually determined. The percentage TUNEL-positive nuclei were calculated in 30 random fields for each group.

##### Statistical Analysis for the TUNEL Assay

Statistical analyses were performed by using TIBCO Statistica version 13.3 (TIBCO Software Inc., Palo Alto, CA, USA). The arithmetical means (Xs), standard deviations (SDs), medians (Mes) and range were calculated. The quantitative values were analyzed for normality using the Shapiro–Wilk test. As most of the distributions deviated from a normal distribution, nonparametric tests were used. To assess the differences between the groups, the non-parametric Kruskal–Wallis test with Dunn’s multiple comparison test for post hoc analysis was used. The level of statistical significance was *p* < 0.05.

## 5. Conclusions

This study shows that immunosuppressive protocols used chronically at therapeutic doses increase the percentage of cells undergoing apoptosis in rat cardiac tissue and thus may contribute to the development of heart failure. The most significant changes were detected when a calcineurin inhibitor was used in combination with mycophenolate mofetil (TMG, CMG). There was a significant increase in caspase 3 in both groups. The TRG group displayed the lowest percentage of apoptotic cells. The intrinsic pathway of apoptosis was confirmed only in the TMG group, where an increase in caspase 9, an increase Bax protein expression and a decrease in Bcl-2 protein expression were noted. These results are an introduction to further research.

### Strengths and Limitations of the Study

The strong side of this study was its experimental nature, where rats were treated with three-drug immunosuppressive regimens consistent with those used in the treatment of organ recipients. Apart from that, the period of 6 months of drug administration corresponded to chronic treatment in humans, covering a period of about 15 years. Unfortunately, the limitations of the experiment stem from the fact that it was conducted on animal tissue, which undoubtedly fails to represent exactly the effects of the immunosuppressive drugs on human tissue.

## Figures and Tables

**Table 1:** Comparison of the percentages of TUNEL-positive cells in cardiomyocytes of rats between the control and experimental groups.

Group	X ± SD	Median (Range)
C	10.7 ± 1.4	10.7 (7.6–14.8)
TRG	16.7 ^b^ ± 2.7	17.0 (12.9–23.0)
CRG	18.5 ^a^ ± 1.6	18.9 (13.9–22.6)
MRG	23.0 ^a^ ± 4.0	21.4 (16.0–30.9)
CMG	25.5 ^a^ ± 4.8	24.2 (18.1–35.8)
TMG	27.9 ^a^ ± 5.1	27.5 (17.8–33.7)

C—control group without any medication; TRG—rats treated with rapamycin, tacrolimus, glucocorticosteroids; CRG—rats treated with rapamycin, cyclosporin A, glucocorticosteroids; MRG—rats treated with rapamycin, mycophenolate mofetil, glucocorticosteroids; CMG—rats treated with cyclosporin A, mycophenolate mofetil and glucocorticosteroids; TMG—rats treated with tacrolimus, mycophenolate mofetil and glucocorticosteroids; X ± SD—arithmetical mean ± standard deviation; ^a^—*p* < 0.001 vs. control; ^b^—*p* = 0.003 vs. control (Kruskal–Wallis test).

## Data Availability

The original contributions presented in the study are included in the article, further inquiries can be directed to the corresponding author.
